# BREAKING THE TABOO OF SERIOUS ILLNESS, DYING, AND LOSS IN THE COMMUNITY: CARING NEIGHBORHOODS IN BELGIUM

**DOI:** 10.1093/geroni/igad104.0392

**Published:** 2023-12-21

## Abstract

Since the first experiments in 2013 until the launch of 133 Caring Neighbourhoods officially supported by the Minister of Welfare in 2022, Caring Neighbourhoods have become in 10 years time a community-wide movement in Flanders and Brussels. Caring Neighbourhoods aim to support older adults to age well in place: where access to care services is guaranteed, where residents know and help each other, where there are opportunities to meet. The presentation uses insights froms 2 project waves. First, results are presented from the evaluation research of 34 Caring Neighbourhoods in 2020. The project coordinator and a volunteer/community member from each project participated in 1 of the 10 focus groups, using participant-generated photo-elicitation to describe and understand the experienced outcomes and mechanisms of their caring neighbourhood. In evaluating projects it is interesting to see what is going on, what they are doing, what their outputs are. But this presentation will focus on what they were not doing. The question will be raised whether caring neighbourhoods are intended to only provide “small care” and “little help”, or to help people with complex needs, in the most difficult moments of life? Second, findings are presented from the current 133 Caring Neighbourhoods and how some of them are trying to respond to issues associated with serious illness, dying, death and loss. The presentation will give insights in actions in how neighbourhoods are encouraged to give more attention to end-of-life topics, and will provide examples on how they are transforming caring neighbourhoods into compassionate neighbourhoods.

